# QIP: Enhancing Staff Awareness of Anti-Ligature Room Allocation in the Clinical Decision Unit

**DOI:** 10.1192/bjo.2025.10346

**Published:** 2025-06-20

**Authors:** Hussain Bux, Alex Olaye, Adam Gadhvi, Suzana Anjum, Bardhan Shah Jung

**Affiliations:** North East London Foundation Trust, London, United Kingdom

## Abstract

**Aims:** Ogura Ward is a psychiatric inpatient Clinical Decision Unit (CDU) which contains 20 rooms for male patients with acute psychiatric illnesses. These patients are frequently at high risk of self-harm. In a bid to minimise the risk of self-harm, Ogura ward contains two anti-ligature rooms designed to keep patients safe. Correct allocation of patients to these rooms is crucial for patient safety. However, gaps in staff awareness regarding the locations of these rooms and the patients assigned to them were identified, emphasising the need for targeted interventions.

This Quality Improvement Project (QIP) aims to improve staff awareness of the anti-ligature room locations and the patients assigned to each room. The project aims to achieve an 80% improvement in staff awareness over two months.

**Methods:** A Quality Improvement Project (QIP) was implemented using a Plan-Do-Study-Act (PDSA) cycle over two months. Initial staff consultations and surveys highlighted gaps in awareness, forming the basis for intervention design. Two interventions were introduced one week apart:

A daily updated poster displayed in the nursing station, identifying the anti-ligature rooms and patients allocated to these.

Structured reminders during multidisciplinary team (MDT) meetings.

After each intervention, a re-survey was conducted to evaluate its effectiveness before the next intervention.

**Results:** The first intervention (poster) resulted in 75% of staff correctly identifying the room locations and 65% identifying the patient allocations up from 23.53% and 41.17% respectively. The second intervention (MDT reminders) further improved awareness, with 88.2% of staff accurately identifying allocations. Staff communication ratings also improved, with 94.11% rating MDT communication as excellent or good, up from 35.29%. Despite progress, out-of-hours patient admissions posed challenges to maintaining up-to-date awareness. These findings emphasised the need for sustainable real-time solutions. Other challenges included ensuring consistency and sustainability of interventions.

**Conclusion:** This QIP successfully enhanced staff awareness and communication regarding anti-ligature room allocations, contributing to improved patient safety practices. The project is currently in its third cycle, focusing on integrating room allocation information into the RIO electronic system to enable real-time accessibility and sustain improvements. Future work will involve completing this integration as well as expanding the project to other wards within the Trust to assess the broader impact and applicability of these interventions.

